# A machine learning model based on readers’ characteristics to predict their performances in reading screening mammograms

**DOI:** 10.1007/s12282-022-01335-3

**Published:** 2022-02-05

**Authors:** Ziba Gandomkar, Sarah J. Lewis, Tong Li, Ernest U. Ekpo, Patrick C. Brennan

**Affiliations:** Image Optimisation and Perception Group (MIOPeG), Discipline of Medical Imaging Sciences, Faculty of Medicine and Health, University of Sydney, Western Ave, Camperdown, Sydney, NSW 2006 Australia

**Keywords:** Area under curve, Inter-observer variability, Machine learning, Mammography, ROC curve

## Abstract

**Objectives:**

Proposing a machine learning model to predict readers’ performances, as measured by the area under the receiver operating characteristics curve (AUC) and lesion sensitivity, using the readers’ characteristics.

**Methods:**

Data were collected from 905 radiologists and breast physicians who completed at least one case-set of 60 mammographic images containing 40 normal and 20 biopsy-proven cancer cases. Nine different case-sets were available. Using a questionnaire, we collected radiologists’ demographic details, such as reading volume and years of experience. These characteristics along with a case set difficulty measure were fed into two ensemble of regression trees to predict the readers’ AUCs and lesion sensitivities. We calculated the Pearson correlation coefficient between the predicted values by the model and the actual AUC and lesion sensitivity. The usefulness of the model to categorize readers as low and high performers based on different criteria was also evaluated. The performances of the models were evaluated using leave-one-out cross-validation.

**Results:**

The Pearson correlation coefficient between the predicted AUC and actual one was 0.60 (*p* < 0.001). The model’s performance for differentiating the reader in the first and fourth quartile based on the AUC values was 0.86 (95% CI 0.83–0.89). The model reached an AUC of 0.91 (95% CI 0.88–0.93) for distinguishing the readers in the first quartile from the fourth one based on the lesion sensitivity.

**Conclusion:**

A machine learning model can be used to categorize readers as high- or low-performing. Such model could be useful for screening programs for designing a targeted quality assurance and optimizing the double reading practice.

**Supplementary Information:**

The online version contains supplementary material available at 10.1007/s12282-022-01335-3.

## Introduction

To improve the quality of screening mammography programs, guidelines have proposed criteria based on the reader characteristics for certification to undertake independent mammography interpretation [1, 2]. Although these guidelines mostly use annual mammographic reading volume as a criterion for certification, there are discrepancies between countries in the volume read required for certification. For example, whilst the USA require 960 reads biannually, European countries and Australia require at least 5000 and 2000 reads, respectively, per year. Moreover, inconsistent relationship between mammography reading volume and sensitivity was reported, with studies showing no association [3–10], positive association [11–15], and quadratic associations [16]. Similarly, mixed findings have been reported for the relationship between the specificity and mammographic reading volume: non-significant [3, 6, 8, 9, 16], positive [4, 14, 15, 17, 18], quadratic [11], and negative associations [5].

Improving understanding about the relationship between readers’ characteristics and mammography interpretation performances could be used to inform targeted quality assurance and surveillance measures for readers, particularly for those at high risk of under-performing. Such programs might improve the performance of the screening program [19].

In previous studies [3–6, 8, 9, 11, 14–18] often low numbers of observers were involved and most of the studies were done using images produced by non-digital mammography units. In the current study, we aim to use data from very large cohort of readers (905 radiologists and breast physicians) and digitally acquired images to assess the relationship between reader characteristics and mammography image interpretation performance. We also propose a machine learning model to predict reader performances using their characteristics.

## Materials and methods

### Image case sets and participants

Using Breast Screen Reader Assessment Strategy (BREAST; http://sydney.edu.au/health-sciences/breastaustralia/) platform. The process of the case selection has been described elsewhere [20]. All cases were selected by a senior breast radiologist based on radiological and pathological reports and contained four digital mammograms (both sides, two views). Each case set included 40 normal (based on a two-year follow-up) and 20 biopsy-proven cancer cases. Cancer cases contained lesions with different mammographic features: malignant masses, calcifications, asymmetries, or architectural distortions. The senior radiologists, who selected the cases in various test sets, also ensured the inclusion of only good quality images. The quality assessment process considered image criteria related to the proper breast positioning, exposure parameters, contrast, and artifacts. Please see the Supplementary Materials for the summary of the criteria.

Table 1 summarises the characteristics of the test sets. Average size of the cancer cases and distribution of cases across various BI-RADS density categories and various cancer types is shown. All cases were retrieved from the screening archive. Information about the presence of benign lesions was not available as benign findings are not routinely collected in the screening archive. Images were acquired using the mammography machines from different manufacturers in use in the screening facilities. Three of the nine image sets included a small (< 5%) to moderate (20%) fraction of computed radiography (CR) cases, but the vast majority were from full-field digital mammography (FFDM) systems.

**Table 1:** Characteristics of the cases in each test set

Feature	Set 1	Set 2	Set 3	Set 4	Set 5	Set 6	Set 7	Set 8	Set 9
Breast density									
BI-RADS A	5	4	6	1	9	5	6	8	6
BI-RADS B	31	20	20	21	35	33	25	30	21
BI-RADS C	23	34	28	37	15	19	23	22	30
BI-RADS D	1	2	6	1	1	3	6	0	3
Cancer type									
Architectural Distortion	2	1	1	4	2	3	0	4	0
Calcification	4	4	5	4	3	2	7	0	2
Non-specific density	4	4	3	4	3	1	2	4	5
Discrete Mass	3	1	6	1	3	1	3	0	4
Spiculated Mass	0	1	3	3	4	3	8	0	4
Stellate	7	9	2	4	5	10	0	12	5
Mean size (mm)	7.4	5.6	5.5	12.8	7.4	9.2	7.3	5.9	6.8
Case difficulty ranking*	9	8	3	7	5	4	1	2	6

*1 is the easiest and 9 is the most difficult test set

Data were collected from 905 certified radiologists and breast physicians who completed at least one of the test sets. Using a questionnaire, we collected radiologists’ demographic details. Participants were recruited in nine different workshops. One test set was allocated to each workshop and made available to the participants. All interested radiologists/breast physicians who attended each workshop were allowed to do the test sets. Table 2 show radiologists’ responses.

**Table 2:** The mean value (standard deviation) of area under receiver operating characteristics curve (AUC), sensitivity, specificity, and lesion sensitivity of participants when grouped by various variables

	No	AUC	Sensitivity	Specificity	Lesion sens
Gender^†^					
Male	144	0.81 (0.10)	77.20 (17.31)	75.48 (17.26)	65.46 (19.80)
Female	184	0.86 (0.07)	82.21 (13.33)	80.22 (14.31)	74.28 (15.82)
Not responded	577	0.83 (0.09)	82.28 (14.45)	73.33 (17.12)	68.76 (18.48)
Position					
Breast physician	39	0.84 (0.07)	81.6 (14.31)	73.31 (17.4)	68.06 (16.47)
Radiologist	866	0.83 (0.09)	81.45 (14.86)	75.15 (16.79)	69.41 (18.47)
*P* value^*^		0.880	0.963	0.544	0.467
# Cases per week					
No cases	10	0.70 (0.08)	62.5 (25.08)	68.5 (32.81)	40.58 (13.17)
< 20	261	0.78 (0.1)	75.53 (16.77)	71.25 (18.51)	59.40 (19.66)
20–50	72	0.84 (0.08)	84.38 (13.16)	70.92 (18.19)	68.92 (16.14)
51–100	113	0.85 (0.07)	81.55 (15.64)	77.27 (14.42)	67.56 (18.15)
101–150	122	0.86 (0.07)	84.93 (12.21)	77.38 (14.91)	75.69 (14.10)
151–200	167	0.85 (0.07)	84.61 (10.52)	76.94 (14.95)	76.18 (13.11)
> 200	157	0.87 (0.06)	85.53 (11.6)	78.05 (15.21)	77.31 (15.14)
Not responded	3	0.8 (0.11)	56.67 (30.14)	92 (12.17)	55.67 (31.02)
*P* value^*^		** < 0.001**	** < 0.001**	** < 0.001**	** < 0.001**
# Hours per week					
None	10	0.70 (0.08)	62.5 (25.08)	68.5 (32.81)	40.58 (13.17)
< 4	307	0.80 (0.1)	77.86 (15.92)	72.75 (18.42)	62.33 (18.98)
5–10	299	0.83 (0.08)	81.33 (14.9)	75.04 (16.07)	70.73 (17.51)
10–15	100	0.86 (0.06)	84.57 (11.12)	77.48 (13.71)	74.84 (14.74)
16–20	82	0.86 (0.08)	84.51 (16)	76.76 (16.05)	74.78 (18.51)
21–30	46	0.89 (0.06)	89.15 (8.32)	79.28 (13.51)	81.40 (9.99)
> 30	61	0.88 (0.06)	85.09 (10.52)	80.28 (13.43)	73.41 (17.01)
*P* value ^*^		** < 0.001**	** < 0.001**	**0.007**	** < 0.001**
Cases in usual practice					
Both	113	0.80 (0.1)	76.74 (15.41)	73.92 (18.62)	61.78 (18.93)
Hard copy	129	0.82 (0.1)	79.96 (15.14)	74.55 (16.89)	67.03 (19.72)
Soft copy	662	0.84 (0.08)	82.55 (14.51)	75.40 (16.48)	71.1 (17.67)
Not responded	1	0.83 (0)	85 (0)	57 (0)	71 (0)
*P* value ^*^		** < 0.001**	** < 0.001**	0.555	** < 0.001**
Screening program					
BreastScreen Aotearoa	181	0.87 (0.06)	87.92 (9.57)	76.09 (13.88)	82.15 (10.53)
BreastScreen Australia	451	0.84 (0.08)	82.19 (13.9)	76.98 (16.14)	69.80 (17.14)
No	273	0.79 (0.09)	75.96 (17.07)	71.25 (18.95)	60.13 (19.2)
*P* value^*^		** < 0.001**	** < 0.001**	**0.007**	** < 0.001**
Fellowship					
Yes	205	0.85 (0.08)	82.22 (13.93)	78.38 (15.7)	73.34 (17.24)
No	574	0.82 (0.09)	80.72 (15.41)	74.15 (17.11)	69.69 (18.67)
Not Responded	126	0.85 (0.07)	83.57 (13.28)	73.88 (16.62)	61.34 (16.41)
*P* value^*^		**0.001**	0.312	**0.001**	**0.015**
Age					
Q1: [28–44)	212	0.81 (0.1)	77.81 (16.54)	74.77 (18.14)	65.65 (19.87)
Q2: [44–54)	234	0.84 (0.08)	83.34 (13.79)	74.46 (17.52)	71.02 (17.85)
Q3: [54–61)	211	0.85 (0.07)	83.65 (12.09)	76.27 (14.29)	72.49 (15.74)
Q4: 61 +	248	0.84 (0.09)	81.38 (15.73)	74.88 (16.86)	68.53 (19.15)
*P* value^*^		** < 0.001**	** < 0.001**	0.932	**0.001**
# Years 1					
Q1: [0–3)	208	0.79 (0.09)	76.8 (16.26)	71.59 (18.98)	60.85 (18.47)
Q2: [3–10)	237	0.83 (0.08)	80.44 (15.76)	76.07 (15.79)	69.09 (18.87)
Q3: [10–18)	210	0.86 (0.07)	84.82 (12.18)	77.2 (16.09)	73.68 (16.4)
Q4:18 +	250	0.85 (0.08)	83.47 (13.61)	75.23 (16.07)	73.05 (17.01)
*P* value^*^		** < 0.001**	** < 0.001**	**0.016**	** < 0.001**
# Years 2					
Q1: [0–1)	224	0.79 (0.09)	75.07 (17.62)	72.98 (19.3)	60.29 (19.11)
Q2: [1–10)	223	0.85 (0.08)	82.28 (14.16)	77.43 (14.83)	72.04 (17.85)
Q3: [10–19)	237	0.85 (0.08)	85.25 (12.16)	74.21 (16.28)	73.94 (16.09)
Q4:19 +	221	0.85 (0.08)	83.07 (13.14)	75.16 (16.47)	70.77 (17.95)
*P* value^*^		** < 0.001**	** < 0.001**	**0.012**	** < 0.001**

The number (No.) of participants (out of 905) and *p* values, indicating whether the difference in each performance metric among categories is significant, are also shown. The reader’s age, number of years reading mammograms (# Years 1), and number of years certified as screening readers (# Years 2) were discretized in four quartiles

†As majority of participants did not provide the response to the questions about their gender, the *p* value for this feature is not reported

*It should be noted that although *p* values are significant, adjustments to confounding factors are required for judging the effect of each variable on the performance

Significant *p* values are in bold

### Experimental protocol

Readings were conducted either at conference venues in rooms carefully designed to match radiologic reporting environments or in the reporting rooms of radiologists’ practicing facilities between January 2012 and January 2019. Half of the assessments (50.28%) were conducted in a conference venue. The radiologists viewed images using Sectra Breast Imaging PACS (Sectra, Linköping, Sweden; Hologic, Bedford, Mass). Options for zooming, panning, and windowing were available to readers. Ambient lightning was below 10 lx in the room. The workstations were linked to either two MFGD 5621 monitors (Barco, Kortrijk, Belgium), or two RadiForce G51 monitors (Eizo, Ishikawa, Japan). All monitors were calibrated to the Digital Imaging and Communication in Medicine gray-scale standard display function, had a contrast ratio of 365:1, and displayed a maximum luminance within 5% of 475 cd. m^−2^, minimum luminance of 1.3 cd.m^−2^.

Each reader reported their findings per case using the Royal Australian and New Zealand College of Radiologists (RANZCR) rating system, which rates mammographic findings into the following five categories: (1) no significant abnormality, (2) Benign, (3) Equivocal, (4) Suspicious, and (5) Malignant. The RANZCR rating system is similar to the Tabar five-tier grading system [21]. Although it is different from Breast imaging-reporting and data system (BI-RADS) classification system [22], the two systems are translatable. Grades 1, 2, and 5 in the RANZCR system is identical to BI-RADS 1, 2, and 5, respectively. The RANZCR grade for the cases graded as BI-RADS 3 and 4A, is “3” while the RANZCR grade equivalent to the BI-RADS 4B and 4C, is “4”. The readers had no prior knowledge about the prevalence and types of cancer included in the case sets. They were also asked to annotate the location of cancer. By using the readers’ ratings, a receiver operating characteristic (ROC) was generated and the area under the ROC curve (AUC) was calculated. All markings rated as 3 or more were considered as positive to compute sensitivity, specificity, and lesion sensitivity.

### Proposed machine learning model

To predict the readers’ AUC values and their lesion sensitivity, radiologists’ characteristics were fed into two ensemble of regression trees (250 trees with surrogate splits to handle the missing data combined using the bagging method). Among all collected characteristics, participants’ gender was excluded as considerable number of participants did not provide response to this question. We selected the ensemble of trees method as the feature selection is embedded within the model and it can successfully handle missing data. The AUC value provides a measure of over-all accuracy and the trade-off between case-level sensitivity and specificity while lesion sensitivity indicates how well radiologists perform in the actual annotation of the cancer location (lesion-level analysis).

We also included the case set difficulty measure. To do so, for each case set we used the jack-knifing free response operating characteristic curve (JAFROC) figure of merit (FOM) [23], which measures the trade-off between lesion sensitivity and specificity. We ranked the case sets from 1 to 9 and used this value as one of the regression model’s inputs (ordinal variable).

We used the average JAFROC FOM from five radiologists, who used a similar platform to read the images. For six sets, readings from all five was available while for three sets, readings from four of these five radiologists were available. All these radiologists read 101–150 mammograms per week, had 10–16 years of experience in reading mammograms, were screen readers, and devoted 10–20 h of their practice to reading breast images.

### Statistical analysis and validation

The mean AUC, sensitivity, specificity, and lesion sensitivity for readers in each group of categorical variables was calculated. Using the Kruskal–Wallis test, we investigated if the performance metrics differed across various values of each variable (Table 2). For participant’s age, number of years reading mammograms, and number of years certified as a screening reader, we calculated the range of each quartile (presented in Table 2) and explored if performance metrics differed significantly in each quartile using the Kruskal–Wallis test. For analysing each variable, the samples where responses were not provided by the reader, were omitted for the Kruskal–Wallis test. We also calculated the adjusted odds ratio (OR) of various factors for having an AUC (and lesion sensitivity) higher than the median value to adjust for confounding effects. In all statistical tests, a *p* value of < 0.05 was considered statistically significant.

We performed the analysis twice. Firstly, we treated work-load variables (hours and case number) as ordinal and considered number of years reading mammograms and number of years certified as a screening reader as ordinal variables. The rest of the variables have been treated as categorical variables in both types of analyses. Secondly, we categorised number of years reading mammograms and number of years certified as a screen reader into four categories (representing four quartiles) and treated all variables as categorical variables. We conducted the same process for the lesion sensitivity. These statistical analyses were conducted in the R (version 3.6.0) environment.

The performances of the models for predicting the AUC and lesion sensitivity were evaluated using leave-one-out cross validation (LOOCV). We calculated the Pearson correlation coefficient between the predicted values by the model and the actual AUC and lesion sensitivity. The model’s output can also be thresholded and used to categorise readers as high-performers and low-performers. We grouped readers as two categories in five different ways based on their performances:

(1) Those in the lowest quartile (low-performers) *versus* those in the second, third, highest quartiles (high-performers).

(2) Those in the lowest and second quartiles (low-performers) *versus* those in the third, highest quartiles (high-performers).

(3) Those in the lowest, second, and third quartiles (low-performers) *versus* those in the highest quartiles (high-performers).

(4) Those in the lowest quartile (low-performers) *versus* those in the highest quartiles (high-performers).

(5) Lowest one-third of the readers (low-performers) *versus* highest one-third of the readers (high-performers).

In each one of the scenarios, we then evaluated the model’s over-all accuracy by generating the ROC curve by applying different cut-off values to the performance metrics predicted by the model. The models were built and cross validated in MATLAB 2018b (Mathworks, MA, US) on a desktop (Dell Precision 5820 Tower). The cross-validation processes for the AUC and lesion sensitivity models completed in 784 and 622 s, respectively.

Currently, in most countries, the interpretive volume serves as the main eligibility criteria for being qualified as a screen reader [2]. Therefore, for all these five scenarios, a model which relies on the reading volume (number of cases per week) and case set difficulty served as the comparison baseline. To build the baseline model, a linear regression model and an ensemble of regression trees were tested. Although the performance of the ensemble was slightly better, the difference was insignificant. For consistency in the comparison, the ensemble of regression trees was used as the baseline comparison. It should be noted that this is slightly more accurate and complicated compared to the simple thresholding of the reading volume.

## Results

Table 2 indicates the performance metrics. As shown, the p-values for all performance metrics were significant for number of cases per week, number of hours per week, being a screen reader, number of years reading mammograms (# Years 1 in Table 2), and number of years certified as screening readers (# Years 2 in Table 2). Although most of the p-values for the last three variables in Table 2 were significant, adjustments against the confounding factors and case set difficulties were required. The last three variables presented in Table 2 were significantly correlated with each other. Therefore, we only included number of years reading mammograms, which resulted in the highest non-adjusted OR for the AUC and Lesion sensitivity. Table 3 shows the characteristics which led to an adjusted OR significantly greater than, or less than 1. As stated, number of hours, reading volume, and years of experience are treated once as ordinal and once as categorical variables. Based on the first analysis presented in Table 3, a one-year increase in years of experience increases the odds of having AUC above the median by 3% (CI 1%–5%; OR of 1.03). Also, a single unit of increase in two ordinal variables, representing seven levels of interpretive hours and volume, increases the odds of having an AUC above the median by 28% (CI 16%-40%; OR of 1.28) and 38% (CI 23%–55%; OR of 1.38), respectively.

**Table 3:** Adjusted Odds Ratios (OR) for readers characteristics, which led to an adjusted OR, significantly greater than, or less than 1

Variables	AUC > Median	L. Sens. > Median
Treating #Hours, #Cases, and #Years as ordinal variables		
Radiologist: breast Phy	2.31 (1.14–4.69)	–
# Hours (ordinal 1–7)	1.28 (1.16–1.40)	1.21 (1.07–1.35)
# Cases (ordinal 1–7)	1.38 (1.23–1.55)	1.35 (1.23–1.48)
# Years	1.03 (1.01–1.05)	–
Treating #Hours, #Cases, and #Years as categorical variables		
Radiologist: breast Phy	2.56 (1.22–5.36)	–
# Year Q3: Q1	2.34 (1.45–3.78)	2.03 (1.24–3.31)
# Year Q4: Q1	1.72 (1.07–2.76)	–
# Cases 21–50: none	13.66 (1.33–140.61)	23.57 (2.38–233.46)
# Cases 51–100: none	12.53 (1.23–127.74)	26.96 (2.75–264.54)
# Cases 101–150: none	21.8 (2.15–221.19)	31.41 (3.24–304.6)
#Cases 151–200: none	–	23.42 (2.43–225.88)
#Cases 201 + : none	15.06 (1.48–152.78)	33.12 (3.4–322.47)

The ORs for having an area under receiver operating characteristics curve (AUC) and lesion sensitivity (L. Sens.) greater than the median values are shown. The analyses have been done twice (Ordinal and Categorical). Only significant values are shown

#Hours, #Cases, and #Years represent number of hours reading mammograms per week, number of mammographic cases per week, and number of years reading mammographic images

“–” represents non-significant adjusted ORs

**Fig. 1:**
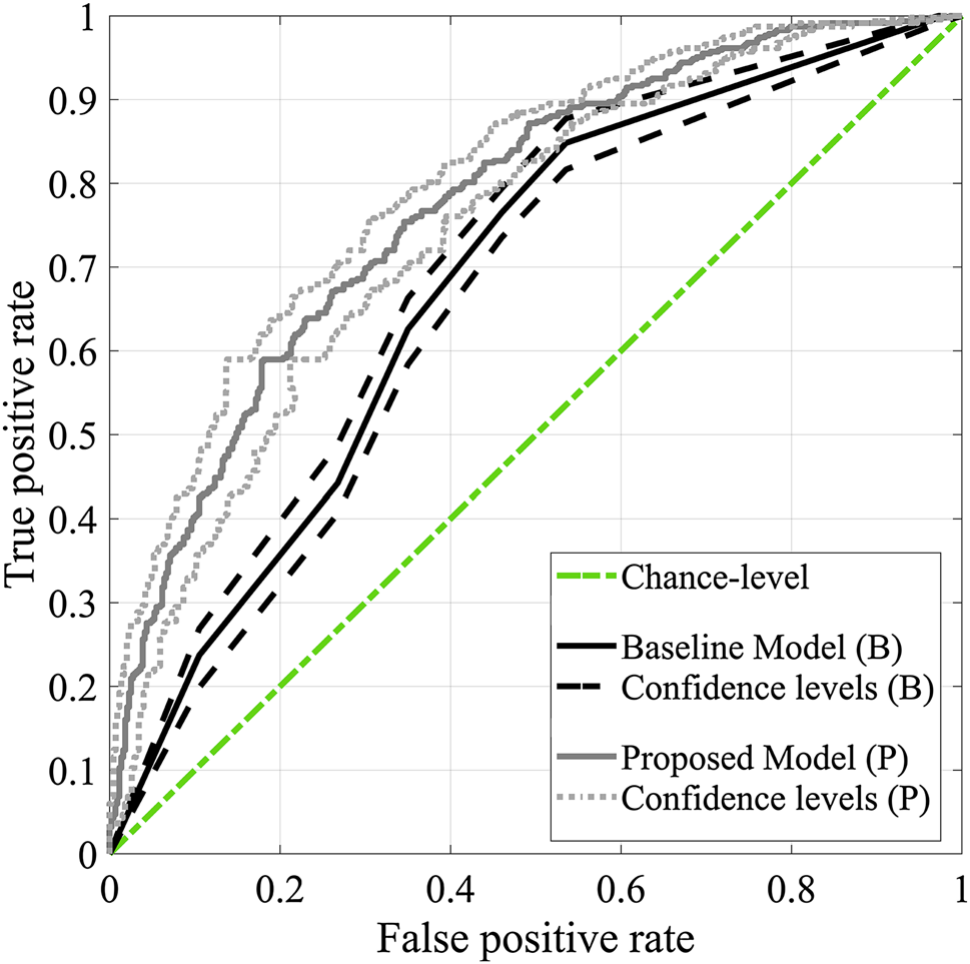
The receiver operating characteristics (ROC) curves and their confidence intervals (dashed and dotted lines) for categorising readers as high- and low-performers using the median AUC value (the second way of categorisation in the Statistical Analysis and Validation section). The grey ROC curve represents the ROC of the proposed ensemble of regression trees and the black ROC curve represents how well one can categorise these two groups of readers if only the number of cases per week (measure of reading volume) and case set difficulty is used

The ROC curves for the proposed method and the baseline comparison method for categorising readers (the second way of categorisation in the Statistical Analysis and Validation section and the second column in Table 4) is shown in Fig. 1. The baseline model represents how well one can categorise these two groups of readers if only the number of cases per week (measure of reading volume) and case set difficulty was used. As indicated in the figure and Table 4, the proposed model outperformed the baseline model for categorising readers.

**Table 4:** The performance of the proposed model’s prediction if used for categorizing the readers as high- and low-performers

	1	2	3	4	5
Predicting over-all reader’s performance, as measured by AUC					
Baseline model	0.72(0.68–0.75)	0.68(0.64–0.71)	0.65(0.62–0.69)	0.75(0.71–0.79)	0.72(0.68–0.76)
Proposed model	0.81(0.79–0.84)	0.78(0.74–0.80)	0.73(0.70–0.77)	0.89(0.86–0.92)	0.85(0.82–0.88)
Predicting reader’s lesion sensitivity					
Baseline model	0.71(0.68–0.75)	0.69(0.66–0.72)	0.68(0.63–0.71)	0.78(0.74–0.82)	0.74(0.71–0.78)
Proposed model	0.81(0.78–0.84)	0.79(0.76–0.81)	0.79(0.75–0.82)	0.91(0.89–0.94)	0.88(0.86–0.91)

The numbers in the columns represent five different ways of categorizing readers as low- and high-performers, as presented in the Statistical Analysis and Validation section. The performance is measured by area under receiver operating characteristics curve (AUC) and corresponding 95% confidence interval for the AUC values. The baseline model for the comparison only includes reading volume (as measured by number of cases per week) and set difficulty as its inputs

The Pearson correlation coefficient between the predicted AUC and the lesion sensitivity by the ensemble models and the actual values was 0.59 (*p* < 0.001) and 0.58 (*p* < 0.001), respectively. Using MATLAB’s *predictorImportance* function, the three most important features in the AUC model were number of years since certification, position, and number of cases per week while the three features for the lesion sensitivity were number of cases per week, number of hours per week, and number of years reading mammograms.

Table 4 shows the AUC values of the model for classifying the readers into high- or low-performers. Column Numbers represent the five different ways of categorizing readers as low- and high-performers, as presented in the Statistical Analysis and Validation section. As screening guidelines mostly use reading volume as their main criteria for selecting the screen-readers, we also calculated the AUC obtained by using reading volume (as measured by number of cases per week) to classify readers. As explained earlier, test set difficulty was also fed into the model.

**Fig. 2:**
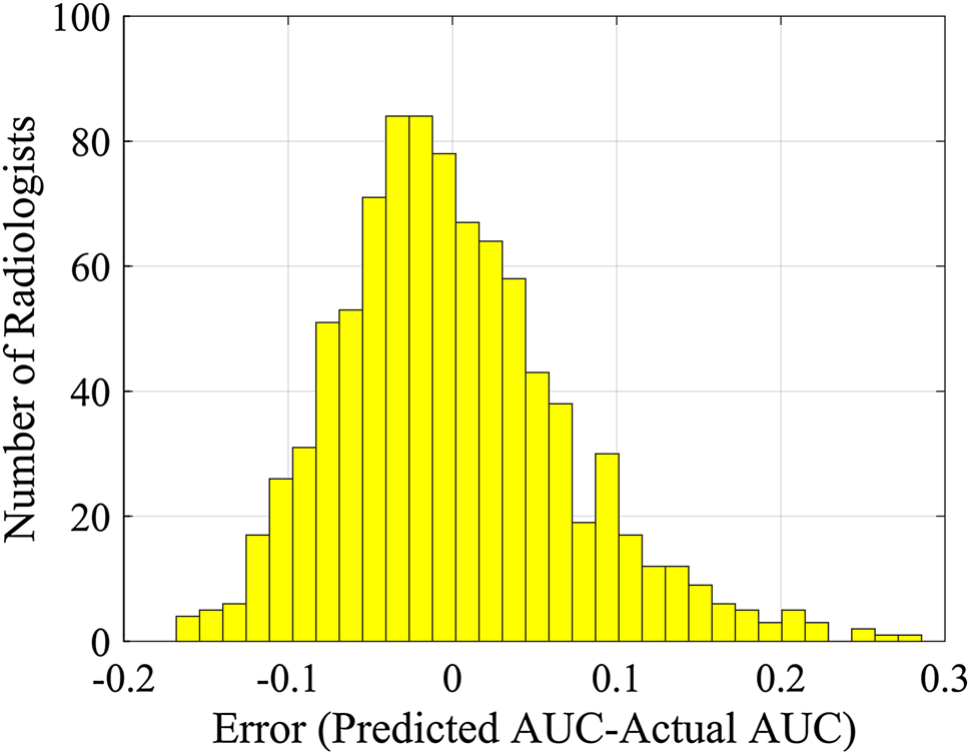
The histogram of the absolute error for predicting the AUC of readers using the proposed machine learning model

The histogram of the model’s absolute error, i.e., the predicted AUC subtracted from the actual AUC, is indicated in Fig. 2. As shown, the absolute error value for most (91%) of the readers is less than 0.1. The error rate (absolute error divided by the value) was less than ± 10% for nearly 87% of readers.

**Fig. 3:**
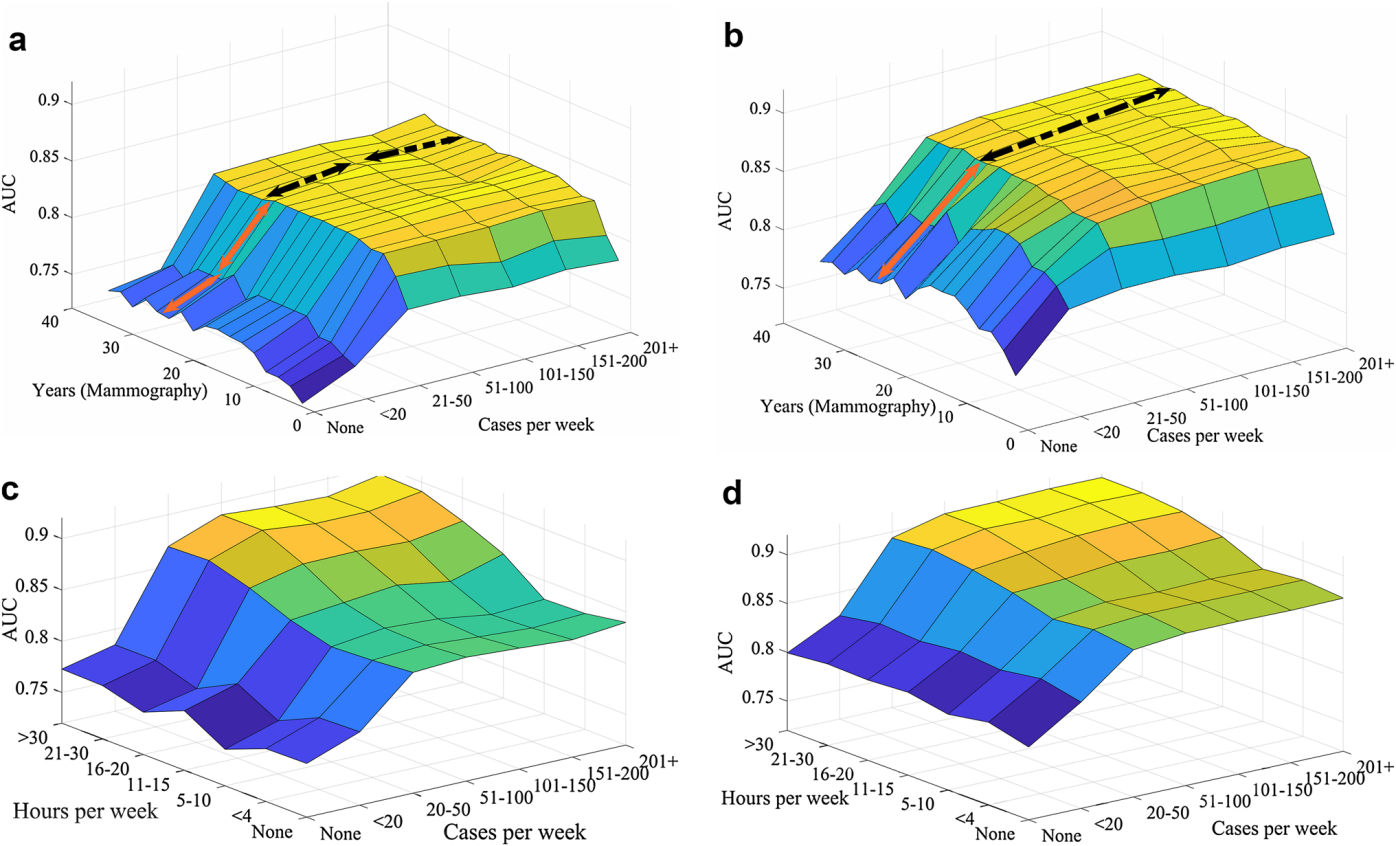
The output of the proposed machine learning model has been simulated for various values for number of cases per week and years of experiences **a** the simulation results for the most difficult; and **b** the simulation result for the easiest case set. The output of the proposed machine learning model has been simulated for all possible pairs for hours per week and cases per week **c** the simulation result for the easiest case set; and **d** the simulation results for the most difficult case set

The proposed machine learning model can be used for analyzing the sensitivity of reader’s performance to each variable. Examples of such analyses is shown in Fig. 3a-d. In Fig. 3a, b, we analysed the model’s output when sweeping the entire grid placed on a two-dimensional feature space including all possible pairs for years of experience in mammography and cases per week. As mentioned earlier, we included a variable to represent the case set difficulty. We simulated the results for the most difficult (a) and the easiest (b) case sets, for a screen reader without fellowship training. These figures indicate how the proposed model can be used to understand readers’ performance in case sets with different levels of difficulties. For example, as shown by the orange arrows in Fig. 3a, for the easiest case set, based on the model’s prediction, readers’ performances increased with a steeper slope from “ < 20” to “21–50” cases per week compared to “None” to “ < 20”. On the other hand, for the most difficult case set the model suggests that the performance of the reader steadily increases from “None” to “21–50” cases per week (orange arrow). From 51 cases per week onwards (dashed black arrows), the trends are only slightly different in two test sets and a peak is evident at 101–151 cases per week only for the most difficult test set.

As another example, we also analysed the model’s output when sweeping the entire grid placed on a two-dimensional feature space including all possible pairs for hours per week and cases per week for a screen reader with fellowship training. The model is simulated for a highly experienced reader (24 years of experiences). We assigned average age and years since certified to this reader, based on average of readers with 20 to 25 years of experience in the original dataset. The simulated resulted are shown in Fig. 3 for the most difficult (c) and the easiest (d) case sets. As shown, the sensitivity of variables to changes of hours per week and cases per week is dependent on how difficult the case set is. For the easiest case set, the performance reach to a plateau for high volume readers (interpretative volume >  = 51) or those spending > 20 h reading mammograms. On the other hand, for the most difficult case set, an increasing trend is exhibited by the model.

## Discussion

This paper investigated how the readers' characteristics affect the performance of readers, using a very large dataset collected from 905 radiologists and breast physicians. Only a few previous studies considered the effect of radiologists’ characteristics on the overall accuracy [7–10, 12–15, 24, 25]. A study in the US [11] found no evidence of a relationship between overall accuracy and larger reading volume while in [4], in concordance with this study, a positive relationship between these two was noted.

We also proposed a model for predicting the reader’s AUC and lesion sensitivity based on reader’s characteristics. The Pearson correlation values suggest a moderate level of correlation between the model’s prediction and the actual performance of the readers. To investigate the usefulness of the model, a baseline model for comparison was created based on the case difficulty and reading volume, as most screening program guidelines use the number of cases as their main criterion for selecting the screening readers. The proposed machine learning model outperformed the baseline model in all various scenarios for categorising high- and low-performing readers.

By having a reliable tool to identify readers at an elevated risk of low performance, the screening programs can establish more frequent and targeted quality assurance schemes for these readers and structure training programs for less experienced radiologists or trainees. Moreover, in settings with limited resources for training or quality assurance, the resources could be more effectively allocated to improve global diagnostic abilities. Feedbacks about radiologists’ decisions [7] and knowledge sharing [26] could improve readers’ performances. Identifying readers at risk of performing lower than the median value and making feedback and knowledge sharing available to them, could improve the overall screening program.

Moreover, our results showed that the proposed machine learning model outperformed the baseline model, which relied on the mammographic reading volume. Currently, the guidelines mostly use annual mammographic reading volume as a criterion for certification. The promising results obtained by the machine learning model, suggest that this model can be used for the certification of the readers. Although, it should be noted that a criterion based on the annual reading volume can be easily understood by the workforce, clinic managers, and policymakers. A more complex machine learning model is difficult to interpret, particularly when someone is classified as unqualified, proper explanations should be provided. Therefore, before using the model, efforts on improving the explainability of the model should be made. Moreover, similar to any other machine learning model, the proposed model might misclassify individuals. The implications of such error on the sensitivity and specificity of a screening program should be investigated. Finally, the feasibility of using this model should be investigated in the context of each screening program considering the availability of the workforce.

Secondly, such a model can be used for pairing low- and high-performing readers in double reading practice. Recent studies showed that more benefits of double reading can be accrued by optimising the pairing [20, 27]. Such strategic matching will lower the chances of pairing low-performing readers and reducing the efficacy of double reading. However, it should be noted that a pre-requisite for translating this into practice is conducting detailed efficacy studies to consider the possible net change in the number of false positives and false negatives by pairing low and high-performing reader, compared to random pairing of the readers. Another important point to consider in the implementation of routinely pairing low- and high-performing readers in double reading practice is the radiology workforce shortage. A shortage of radiologists specialising in Breast imaging is a recognized worldwide challenge [28–30] and the limited number of radiologists in a practice might hinder efforts for optimally pairing readers. To some extent, the wider utilization of teleradiology might help in adopting this pairing strategy, especially where there are significant workforce shortages. Therefore, beside cost-efficacy analysis, the available radiology workforce and infrastructure for teleradiology should be taken account.

This study had a few limitations. Firstly, the prevalence of cancer cases in the case sets was different from the clinical practice. Mixed evidence about the prevalence effect has been reported [31], with studies showing both measurable performance decreases [32–34] and no change in performance [35]. A laboratory effect could also be a limitation [36]. Although it certainly affects our data, a significant positive correlation between laboratory and real practice performance was reported in a previous study [37]. As our model aims at classifying readers into high- and low-performers, the presence of such a correlation implies that our model could predict the performance of readers in real clinical practice to some extent. Moreover, approximately half of the assessments were conducted in the participants’ workplaces and we were not able to ensure that ambient light and monitor’s parameters were in concordance with the assessments conducted in the conferences. Also, although in none of the previous studies such a large cohort of radiologists were included, number of readers in some categories were limited.

As stated, to adjust for the case difficulty, we added a variable ranged from 1 to 9 to show the difficulty of cases in a set. As we analysed the data retrospectively, we could not match the test sets based on their difficulty level. This is another limitation of our study. The difficulty measure was calculated based on assessments from five radiologists, who were included in the analysis. This could cause bias in the performance of the model. However, as these five readers comprised less than 0.56% of our samples, it is reasonable to assume that considerable bias was not introduced to the results.

Currently, among all input variables, only the test set difficulty describes the characteristics of the cases in our test sets. The quality of images produced by the CR and FFDM units are different, and these differences could lead to a change in a reader’s performance [37]. Thus, for a future work, variables describing the mammography unit manufacturer and technology can also be fed as input variables to the performance prediction model to improve its accuracy. In the current study, although images were acquired from various mammography, for all test sets except one, the proportion of CR images was small or non-existent. Hence, our dataset did not provide sufficient power to include mammography technology as one of the inputs. Moreover, as stated in the Materials and Methods section, the senior radiologists, who selected the cases, ensured that only good quality images were included in the test sets. As all images had acceptable quality, a possible effect that different technologies and image processing methods may have on the visibility of cancer was potentially mitigated in our dataset. To explore the effect of adding variables describing the above-mentioned case characteristics on the accuracy of the model, a more diverse set of images should be used to create test sets in the future.

A more comprehensive reader characteristics can also be collected from readers and fed into the model to improve the accuracy of the model. For example, whether working full-time or part-time [11] or being affiliated with academic centres [38] could affect the performance. The current study is a proof-of-concept study and although the proposed machine learning model outperformed the baseline model for categorising high- and low-performing readers, the added benefits of the variables describing additional case and reader characteristics can be explored in the future works.

## Supplementary Information

Below is the link to the electronic supplementary material.

Supplementary file1 (DOCX 15 KB)

## Abbreviations

BREAST: Breast screen reader assessment strategy
ROC: Receiver operating characteristics
AUC: Area under ROC curve
OR: Adjusted odds ratio
LOOCV: Leave-one-out cross validation
JAFROC: Jackknifing free response operating characteristic curve
FOM: Figure of merit
