# Supplementary material for: A machine learning model based on readers’ characteristics to predict their performances in reading screening mammograms
Source: Breast Cancer. 2022 Feb 5;29(4):589–98. doi: 10.1007/s12282-022-01335-3 (PMC9226081; doi:10.1007/s12282-022-01335-3)
Supplement: Supplementary file 1 — Supplementary file1 (DOCX 15 KB) [file 12282_2022_1335_MOESM1_ESM.docx]

**Supplementary Materials**

**Criteria for the quality assessment of MLO (medio-lateral oblique) and CC (cranio-caudal) projections based on the EU requirements [1] and ACR image evaluation system [2] and the review conducted by Lie et al. [3]**

**Positioning**

- The inferior aspect of the pectoral muscle on MLO projection should come to the posterior nipple line and the pectoralis muscle should also be sufficiently wide. Pectoral muscle is at correct angle on MLO projection and visually sharp reproduction of pectoral muscle is seen at image margin on CC projection.

- Inframammary angle visualised on MLO projection and visually sharp reproduction of retro-glandular fat tissue on CC projection

- Visually sharp reproduction of cranio-lateral glandular tissue on MLO projection and visually sharp reproduction of medial breast tissue on CC projection

- Visually sharp reproduction of retro-glandular fat tissue on MLO projection and Visually sharp reproduction of lateral glandular tissue on CC projection

- Nipple in full profile; no skinfolds seen; no sagging. The posterior nipple line on the CC view is within 1 cm of its length on the MLO view.

- Symmetrical images of left and right breast

**Exposure parameters and image details**

- Reproduction of vascular structures seen through most dense parenchyma

- Visually sharp reproduction of all vessels, fibrous strands, pectoral muscle margin, and skin structure (rosettes from pores) along the pectoralis muscle

- Desirable level of breast compression; this can be determined based on how much the breast markings spread out

- Desirable level of exposure can be identified by a better penetration of the denser fibroglandular tissue. In the case of underexposure of the pectoralis muscle, underlying structures in the breast may not be visible.

- Good image contrast, which permits differentiation of subtle tissue density differences.

- Margins of normal breast structures shall be distinct and not blurred

- Low level of noise (noise results in an inhomogeneity in the background) and artifacts (i.e., any density variation on an image that does not reflect true attenuation differences in the tissue)

**References**

[1] E. Commission, “European Guidelines on Quality Criteria for Diagnostic Radiographic Images,” *EUR 16260*, 1996.

[2] V. Stickland, "Mammography Quality Control Manual: Americal College of Radiology 1992 Price: $75.00 (Technologist Manual price $20.00)," Churchill Livingstone, 1994.

[3] Y. Li, A. Poulos, D. McLean, and M. J. E. j. o. r. Rickard, “A review of methods of clinical image quality evaluation in mammography,” vol. 74, no. 3, pp. e122-e131, 2010.
